# Clinical simulation on intimate partner violence in adolescent girls: contributions to health teaching*

**DOI:** 10.1590/1518-8345.7441.4470

**Published:** 2025-02-17

**Authors:** Daniella Yamada Baragatti, Leticia da Silva Scotto, Claudia Adão Alves, Ana Paula de Miranda Araújo Soares, Fernanda Berchelli Girão, Diene Monique Carlos

**Affiliations:** 1 Universidade Federal de São Carlos, Departamento de Enfermagem, São Carlos, SP, Brazil; 2 Scholarship holder at the Conselho Nacional de Desenvolvimento Científico e Tecnológico (CNPq), Brazil; 3 Universidade Federal de São Carlos, Departamento de Medicina, São Carlos, SP, Brazil; 4 Universidade Federal de São Carlos, São Carlos, SP, Brazil; 5 Scholarship holder at the Coordenação de Aperfeiçoamento de Pessoal de Nível Superior (CAPES), Brazil; 6 Universidade de São Paulo, Escola de Enfermagem de Ribeirão Preto, PAHO/WHO Collaborating Centre for Nursing Research Development, Ribeirão Preto, SP, Brazil

**Keywords:** Intimate Partner Violence, Gender-Based Violence, Domestic Violence, Adolescent, Patient Simulation, Primary Health Care

## Abstract

**Objective:**

to learn about the contributions of using clinical simulation with undergraduate health students to care for adolescent girls in situations of intimate partner violence in the context of Primary Health Care.

**Method:**

a qualitative study with 30 medical and 28 nursing undergraduates from a public university in Brazil. Data collection was mediated by holistic debriefing, with subsequent reflective and inductive thematic analysis.

**Results:**

two themes emerged: the complexity of violence and the skills developed using simulation. The students brought up the complexity of care due to family aspects, the particularities of the population, the ambivalence of feelings in a violent relationship, and the limit between preserving autonomy and the obligation to report. Various skills were developed and practiced, such as conflict mediation skills, respect, bonding, welcoming, and recognizing the support network.

**Conclusion:**

the use of simulation proved to be a powerful tool for the teaching-learning process in undergraduate courses, as it allowed reflection on the specificities of adolescent care. The qualitative look at the process also allowed us to delve into how this strategy can be coherent with complex themes that involve the acquisition and experimentation of cognitive, procedural, and attitudinal skills.

## Highlights

**(1)** Simulation proved to be a powerful tool for teaching health courses. **(2)** The qualitative approach allowed for in-depth contributions to a complex subject. **(3)** The students developed and practiced skills for handling a sensitive subject. **(4)** Attributes of Primary Health Care were mobilized in the process. **(5)** The holistic debriefing allowed for an original and pertinent construction of the theme.

## Introduction

Intimate Partner Violence (IPV) is the most common form of violence experienced by women globally. It is any behavior by current or former intimate partners against women that causes physical, psychological, or sexual harm, including physical violence, emotional abuse, rape, as well as controlling and coercive behavior^(1)^.

Teenage girls can also be exposed to this type of violence, known as “Teen dating violence” or “Dating violence”. This type of violence is peculiar to adolescence and can occur in person or by electronic means, between casual or continuous partners^(2)^. Violence between teenage intimate partners has a profound impact on health, opportunities, and well-being throughout life, and can start at an early age and last a lifetime. Young women affected by this type of violence are more prone to depression, anxiety symptoms, use of cigarettes, alcohol, and other drugs, involvement in criminal acts, bullying, and suicidal behavior^(3)^.

IPV is rooted in gender issues, which are social roles assigned to people. Gender constructions are unequal, and social normative instructions grant power to the male^(4)^, which can result in the naturalization of violence in an intimate relationship.

Worldwide, 24% of young women between the ages of 15 and 19 reported having suffered physical and/or sexual violence from an intimate partner throughout their lives, with 16% reporting this type of violence in the previous year. In Brazil, 17% of young women reported the same violence throughout their lives and 10% in the previous year^(5)^.

In general, the main point of care for IPV situations is Primary Health Care (PHC). PHC is the first level of health care and the main gateway to care in Brazil’s Unified Health System (SUS, in its Portuguese acronym). It should provide comprehensive care that has a positive impact on the health situation of communities and is guided by various health policies that highlight the importance of combating situations of violence^(6)^. To this end, the entire process of professional training in health must involve teaching strategies that enable people to experience and reflect on the issue which should happen from the time they graduate.

Clinical simulation is one of the teaching strategies that can be used to work with students and health professionals on IPV among adolescents. Based on the experience of the participants, it is a methodology that uses one or more strategies to promote, improve or validate competencies. Competence is understood as the process of acquiring and building knowledge, skills and attitudes in a social, cultural, historical, and political context^(7)^.

A systematic review found that clinical simulation was identified as an effective educational method for authentic learning, in which undergraduate health students cultivated their knowledge and motivation to learn^(8)^. Despite this conclusion, a recent literature review showed that most simulation scenarios validated in Brazil deal with topics such as urgency and emergency, maternal care and stomatherapy, justifying the focus on complex events such as violence^(9)^. Despite being prevalent in clinical practice, they are rarely addressed in training, even with traditional methodologies^(2)^.

One of the stages of a clinical simulation is debriefing, which takes place immediately after the experience of this strategy. Seeking to promote reflective learning in simulation, researchers developed a holistic debriefing guide that included formative and summative aspects to help educators conduct this important stage^(10)^.

Thus, considering the relevance of nurses and doctors in health services and the importance of the topic being worked on in undergraduate courses, this study sought to learn about the contributions of the use of clinical simulation with undergraduate health students, for the care of adolescent girls in situations of intimate partner violence in the context of Primary Health Care. It is thought that this approach is in line with the prospects for strengthening nursing and health work to meet the needs of adolescents, especially in the context of PHC, through cultural and gender-sensitive actions. Furthermore, it can provide a tool to instrumentalize professors and nurses in the health care of this population^(11)^.

## Method

### Type or design of the study

This was a descriptive-exploratory study with a qualitative approach^(12)^, which followed the conceptual framework of holistic debriefing^(10)^. The aspects contained in the Consolidated Criteria for Reporting Qualitative Research (COREQ) guided the design and presentation of this investigation.

### Study site and setting

The study site was a public university located in the interior of the state of São Paulo, Brazil, which has 254,857 inhabitants in a territorial area of 1,137 km^2^. The university has 64 undergraduate courses, and 15,518 students were enrolled in 2023. The sample was chosen for the researchers’ convenience (they already had contact with the professors and students at this university) and because the research institution uses simulation strategies in its undergraduate programs.

### Period of data collection

Data collection took place between August and October 2023.

### Selection criteria and participants

The inclusion criteria for the participants were: to be an undergraduate medical or nursing student at the mentioned university; to be 18 years or older; to have had contact with theoretical subjects that dealt with violence or the care of children, adolescents and women; to show up on the agreed day and time for the simulation activity and to take part in all the proposed stages. The exclusion criterion was being away from university activities for any reason.

In order to select the participants, the research was supported by the University’s Social Communication Coordination, through emails sent to the course coordinators, as well as by electronic publication on the platforms and social networks of the University’s Academic Centers. There was no contact with the researchers at this stage.

Participants were those who contacted the researcher and showed up on the days scheduled for the simulation, at the Health Simulation Unit, which has simulated environments that are very similar to PHC centers. All the students who showed up agreed to take part in the research.

### Data collection

The clinical simulation was guided by the scenario “Intimate partner violence against adolescent girls in Primary Health Care”, based on Bloom’s Taxonomy for constructing the objectives of educational processes based on cognitive, procedural and attitudinal domains; the standards of practice recommended for simulation design by the International Nursing Association for Clinical Simulation and Learning^(13)^; and the script for constructing a simulated scenario^(14)^. The guide “Three stages of holistic debriefing”^(10)^ was used to finalize the scenario. The scenario was developed and validated by 26 judges specializing in clinical simulation and violence.

The simulations with medical and nursing students took place separately and the overall learning objective of the scenario was for the student to have, by the end of the activity, the ability to develop care for an adolescent woman in a situation of IPV.

The pre-briefing took place by sending an information booklet on violence against women^(15)^ and an infographic on IPV among adolescents^(3)^. The briefing, which lasted between 10 and 15 min, began with a presentation of the environment, equipment, resources, a presentation of the facilitators, information for the students on the learning objective to be achieved in the simulated scenario, as well as the work contract, such as mutual respect, confidentiality and ethics. The students were given an informed consent form to be signed, answered a questionnaire about their socio-economic profile and a description of the clinical case, which was read out to the group. On the day of the clinical simulation, the students were informed about the training of the researchers and the reason for carrying out the work. In addition, if they did not agree to take part in the research, they could still take part in the simulation.

Next, a pair of students were chosen to assist the adolescent in a situation of violence to take part in the scenario, where their mother was also present, with a maximum running time of 30 min. The other students remained as spectators of the scene in a separate space in the simulation unit itself.

The scenarios were managed by two cisgender female facilitators, both nurses and researchers at a public university, one a post-doctoral student and the other a professor. Both experts on the subject had a flowchart to help them conduct the activities, in order to achieve the learning objectives initially proposed. No other people took part in the research scenario apart from those described - facilitating researchers, students and actors.

The debriefing was recorded using a voice recording application for cell phones and transcribed manually. The discussion was conducted using an instrument developed by the researchers, based on the “Three stages of holistic debriefing”^(10)^, namely: (1) Focused (students’ immediate self-reflection after the practical experience), with the following questions: “How do you feel about the simulation experience? What did you learn from taking part in the simulation experience? What skills do you think you developed during the simulation experience?”; (2) Formative (continuous debriefing with the larger group throughout the simulation) with the questions: “As a group, how did you feel during the simulation? As a group, what do you think you know/understand best now? As a group, do you think this simulation experience and discussion helped develop psychomotor skills? Why or why not? What did you learn during this simulation that is similar to what you learned in your undergraduate course? What is different?”; and (3) Summative (final reflection with the larger group and its application to the learning obtained), with the questions: “How did you feel after the simulation experience? How did you perceive the learning progression through this experience? What kind of skills do you think you developed through simulation experience? Do you think that the experience and concepts of violence against adolescents that we discussed in the pre-briefing are clear to you, based on the evidence of best practice? Please explain your answer. Do you think the simulation activity contributed to your professional performance when dealing with adolescents in situations of intimate partner violence?”.

An initial pilot test was carried out to check the suitability of the data collection instrument, and no changes were necessary; the data from the pilot test was incorporated into the research. There were no repetitions of the groups and data saturation, i.e., the deepening of the elements that answered the research questions, was reached in the fifth focus group, with two more already scheduled. Immediately after the clinical simulation, the main researcher took notes on the simulations in a field diary, which helped in the discussion of the findings.

In total, there were seven simulations with groups of approximately eight students, with the participation of 28 nursing undergraduates and 30 medical undergraduates. Taking all the stages into account, the shortest activity lasted 2h35min and the longest 2h55min.

For the presentation of the data, the participants were identified according to the course to which they belonged, Med for Medicine and Enf (from *enfermagem*, which is nursing in Portuguese) for Nursing, followed by a sequential number, according to the order in which they spoke during the activity.

### Data processing and analysis

The data on the participants’ profile, collected through the questionnaire, was presented using descriptive statistics. The debriefing data was transcribed in full using the Google Documents application and analyzed using the reflexive and inductive thematic analysis technique, i.e., emerging from the data^(16)^. Sixteen initial codes and five intermediate codes were generated (“The complexity of violence”, “Feelings and emotions involved in care”, “Importance of welcoming and bonding”, “Specificities in caring for cases of violence against adolescents”, and “Skills developed through the use of simulation”), which in turn were grouped into two final topics.

In order to make this process more reliable, the following criteria were followed: (1) use of a field diary, which allowed for careful and complete recording of the collection process and inferences in the field; (2) construction of the codes and themes between two researchers (1st and 2nd author), agreed by a third (last author); (3) checking the interpreted data with the participants to check for meaning or the need for new insertions - 17 feedbacks by e-mail were carried out, with no new insertions.

### Ethical aspects

The research was approved by the Research Ethics Committee of *Universidade Federal de São Carlos*, under CAAE 63438122.1.0000.5504.

## Results

Of the 58 students, the majority were aged between 20 and 25 (medicine n=25, 83%; nursing n=21, 75%). As for color, the majority declared themselves white (medicine n=22, 73%; nursing n=19, 68%). All were cisgender, with 17 of the medical students declaring themselves as cis men (57%) and 13 (43%) as cis women. Among the nursing students, three (11%) declared themselves as cis men and 25 (89%) as cis women.

After analyzing the qualitative data, the results were divided into two final themes: “The complexity of violence” and “Skills developed through the use of simulation”.

### The complexity of violence

This theme addressed the complexity of elements involved in dealing with cases of IPV against adolescent girls. The first aspect was related to the presentation of the situation - it encompasses broader family aspects that need to be understood in order to provide comprehensive care. The presence of the mother at the initial consultation, as well as family conflicts, including suspicions of domestic violence, made the approach more challenging:

*And also explore the issue of the father’s drinking, which is perhaps a question of an alcoholic in the family environment, so it would be possible to seek out a bit more of this information as well.* (Med 28)

*Then I’d also be interested in talking only to the mother, there’s the whole family issue, the family environment, because she runs away from that environment. It wouldn’t be nice to talk about it in front of her.* (Enf 5)

*There are two people active in the meeting, with different stories. So sometimes when we provide care, it’s just one person or the other person, when there are two, they are complementing the other’s story. This is the first time we’ve had two people with different views on the same subject.* (Med 21)

*I found it very complex, because there’s the issue of the father, I didn’t know if I could ask “did your father beat you? Has there been other violent acts?” Or whether she witnesses other violence with her mother [...] there’s the issue of the boyfriend, there are many complex issues in just one being.* (Enf 1)

As shown in the last statement, the particularities of experiencing IPV in adolescence were revealed. The participants found it more difficult to approach and provide care, given that the adolescent came to the unit not because of an explicit need.

*She’s a teenager and she’s a bit closed off.* (Med 5)

*She didn’t want to be here, she didn’t want to be at the meeting, she was forced by her mother, so she was already disinterested in everything. She was here because her mother made her.* (Enf 8)

The ambivalence in the relationship, being in love and a more veiled experience of violence - given by jealousy and control, emerged in the reports as challenging aspects.

*She didn’t see herself in a situation of violence either. So that was much more challenging.* (Enf 17)

*Because we have to remember the question of maturity, of development. So, at her age, she doesn’t yet have enough developmental maturity to fully understand and judge what is right and wrong.* (Med 4)

*At first, she said she missed going out with her friends, that her boyfriend forbade it and blackmailed her, and then when we asked her about her support network, she said she only had her boyfriend, and the rest was all fake.* (Enf 15)

*And from the looks of it, she’s a very closed off person, right down to the way she sat and so on. So, it’s very difficult to come up with an idea, saying something, when she trusts her boyfriend so much, not least because she can’t deal with her own parents.* (Med 6)

It can be seen from the last statement that the participants had difficulties in building a dialog with an adolescent profile, which is often less verbally elaborate on first contact. The lack of contact with adolescents during their academic career in the health services brought greater challenges in establishing a relationship:

*It’s much more difficult, I think, for us to establish a bond with adolescents, who sometimes only have more contact with their parents, with their friends, right?* (Enf 19)

*I found it a bit more complicated to deal with, because now we’re dealing with a teenager, something we’ve never dealt with before.* (Med 12)

The issue of “being underage” recurred in the discussion of the situation experienced, especially due to doubts about legal reporting, maintaining secrecy and the bond, the power relations established between the teenager and the adult and the best handling of the case:

*I had a lot of doubts about this, about how to say that it wasn’t normal for a 13-year-old child to have a relationship with an adult in his twenties. I wouldn’t know how to put it. Isn’t that a crime?* (Med 2)

*In this case, would it just be the council, or would there be another body to contact? Maybe school? How does the Women’s Police Station communicate with this network?* (Med 6)

*This issue of jealousy, right, is the difference in the power relationship between a 21-year-old adult and a 13-year-old teenager. And there are even a few other things I’ve mentioned, like whether she’s afraid to go against her boyfriend, because I wanted to explore more this balance of power.* (Enf 17)

*Talking more with the patient, making her understand that this is a problem, not talking about the complaint straight away, otherwise she’ll never come back.* (Enf 7)

### Skills developed through the use of simulation

In this topic, the skills that students acquired or practiced through simulation emerged in terms of knowledge, skills and attitudes. Because of the particular purpose of the simulation, it is important to note that dealing with violence, especially against adolescents, is seen as a “*tense, heavy subject* (Enf 12)”, which brings up feelings that need to be talked about and dealt with:

*To be honest, I felt a lot of discomfort. Because it was a very common situation, which reminded me of mothers who were friends of mine, who I saw the way they talked and everything, what they go through, how they feel, so it brings that discomfort and those memories.* (Enf 23)

*So, it distresses me to experience a case of violence, especially with her being an underage teenager.* (Med 3)

*I think it’s very difficult, I even refused to do the simulation because I didn’t feel comfortable. And it’s not a question of understanding and acceptance, I don’t feel comfortable doing it. So, I prefer to observe, to see how people do it to try to learn and get something out of it.* (Med 7)

The last statement demonstrates the coherence of using the simulation strategy to facilitate the teaching-learning process, making the topic more viable and palatable. In this sense, the participants reported mobilizing knowledge they had already acquired, such as the importance of welcoming, bonding and the continuity of care, essential attributes of PHC, as well as articulating theoretical aspects of violence and the support network:

*I think that for the first consultation we had to welcome, to show that it is a safe environment and that we were there to listen without judgment.* (Enf 2)

*She created a bond with the patients, so that she didn’t accuse anyone or make anyone feel bad, in order to provide continuity of care. I think that’s the worst thing about PHC: you scare the patient away at the first visit and they don’t come back.* (Med 14)

*One thing I ended up coming back to is that when we were studying family and domestic violence, people who have lived in a home where there is violence have a greater tendency to perpetuate this violent scenario. And that’s what happened in this case: the mother came from a violent family, she lives in a violent environment, and apparently her daughter is also entering a violent environment. So, I believe that exploring how the daughter sees her mother’s case can help her reflect on how she too is entering a situation, perhaps similar, and can lead to the same future.* (Enf 6)

*Find out whether she has any friends, any other family members she’s close to, to try and see what their support network is. With more time, perhaps as a form of bonding, refer her to a psychologist at the unit, a therapy group. Why doesn’t she have any friends at school? What happened, if something happened, if her boyfriend keeps her away from these friendships.* (Med 15)

In this sense, the students were able to practice caring for IPV among adolescents in PHC; aspects revealed for this skill were a comprehensive view, beyond the signs and symptoms present, active listening and conflict mediation. Attitudinal aspects, without a “recipe”, such as respect and tone of voice were mentioned:

*I think there’s another point I forgot to mention, which is active listening. So, the teenager talking, you are paying attention to what she’s saying, looking at her [...]. I’ve already read in some books that the fact that you keep quiet for a little while makes the person say something new that they might not say if you interrupted them with a question at that moment.* (Med 9)

*Well, I think it’s a very challenging thing, because sometimes we worry about other things that we’re trying to identify, and we end up overlooking these signs and whether there’s anything extra going on.* (Enf 1)

*What I realized is that I need to learn how to approach this case, the way to speak, the tone of voice, I still have a bit of difficulty handling these cases.* (Enf 2)

With the possibility of simulating a service, care for IPV against adolescent girls in PHC was understood as a process that requires light technologies, time and extended actions. This element facilitated discussions about the reporting process, as well as the balance needed in these situations:

*It’s a complicated situation, right, you can’t say to her: “look, break up with your boyfriend, because...”, you can’t say that. I think the safest way to do this would be for her to understand the repercussions of this relationship on her life, and for that to happen it would be a process, it wouldn’t be in one consultation, it would be a long time of talking, both with the health professionals and with the mother and father*. (Med 4)

*This is a skill I have to develop, because I want to know everything about the patient right away. I already think “get out of this, woman, you’re being oppressed”.* (Enf 12)

Finally, the participants brought up the relevance of the simulation, placing them as active people in the construction of the teaching-learning process. Debriefing was pointed out as an important stage in the elaboration of the meaning learned, as well as the acquisition of new skills through meaningful learning:

*I think the simulation is cool, because we see the theory, the signs, and you make it easy for us to put almost all of them in. So, the person avoids speaking, they don’t look at your face to answer you, the conflict that could happen between the girl and her mother, in short. This is good for us because it emphasizes that it is going to happen, we don’t just read about the theory.* (Enf 16)

*So, I think the simulation helped us understand the adolescents, their anxieties, their demands and everything else.* (Med 27)

*I also thought it was great that we were able to ask questions, because as we had mentioned, this issue of compulsory notification or reporting, leaving a record of this, we hadn’t seen this in depth, I thought it was interesting.* (Med 7)

## Discussion

The students realized the complexity of dealing with cases of IPV against adolescent girls in the context of PHC due to broad family aspects; the presence of the mother during the process; the particularities of IPV in adolescence; the difficulty of dialogue during this period; the perception of the ambivalence of feelings in a violent relationship and the balance between preserving the autonomy of the adolescent person and the obligation to report the offense. Despite the complexity of having to deal with the distressing feelings aroused by the event, the students developed various skills, such as conflict mediation skills, and attitudes, such as respect, identifying that IPV care is established as a process. In order to develop it, PHC attributes were mobilized, such as the need for continuity of care through bonding, welcoming, and recognition of the support network.

The sociodemographic profile of the participants corroborates a study in the area^(17)^, in which the majority of undergraduate health students are aged up to 25, white and cis women. Caring for adolescents in situations of violence has many specificities^(3)^. During the simulation, uncertainties regarding the difference between notification and reporting and the limits of the adolescent’s autonomy recurred. All cases of violence are compulsorily notifiable – it is a compulsory communication to the health authority, carried out by professionals, about the occurrence of suspected or confirmed specific health issues, which allows data to be analyzed and can provide support for decision-making^(18)^. This aspect is different from reporting and even articulating care for adolescents, which is sometimes neglected. Considering adolescent care beyond conventional health prisms (based on risk and protection factors), and including them as socially active people, especially vulnerable groups, is seen as a driving force for significant participation in accelerated global action for the health of this population^(19)^.

In Brazil, according to Law 8069 on the Statute of the Child and Adolescent (ECA in its Portuguese acronym), adolescents are subjects of rights who can be cared for autonomously in health services^(20)^. With this in mind, the students consulted the adolescent about whether or not she wanted her mother to leave to listen to her alone during the simulation. Furthermore, although there is nothing specific in the ECA prohibiting dating between an adolescent and an adult, Article 217-A of the Brazilian Penal Code makes it a crime to commit carnal conjunction or a libidinous act with a person under the age of 14, which carries a penalty of imprisonment of between eight and 15 years^(21)^. According to the ECA, it is mandatory to report suspected or confirmed violence against children and adolescents to the Guardianship Council. Although reporting is obligatory, as a crime may be taking place, during PHC care, it is necessary to reflect on this process, including with the adolescent, as well as establishing extended care for her. It is necessary to understand the family dynamic, making the adolescent the center of this care, in line with the ECA and other legislation^(20-22)^.

Still, regarding the care provided in the simulation, the participants reported concerns about how to approach the adolescent and whether they could question her directly about violence. Researchers at an American university have drawn up a guide to help nursing students recognize and help adolescents who are suffering from dating violence. They highlighted the importance of asking directly about this fact, as many adolescents are afraid to talk about these problems or may not perceive their partner’s behavior as violent^(23)^.

A study analyzed undergraduate nursing curricula at public institutions in Brazil in relation to the approach to violence against women^(24)^. It was identified that the approach to adolescent health refers more to aspects of violation of sexual and reproductive rights, with concern related to teenage pregnancy^(24)^. This finding reinforces the importance of simulating violence against an adolescent woman for undergraduates since it is an approach that goes beyond the biological aspects related to violence and is little developed in undergraduate curricula. This is also in line with the importance of this topic for preventing future gender-based violence.

Considering the importance of preparing students, especially in medicine, nursing and obstetrics, to care for women in situations of violence, the World Health Organization (WHO)^(22)^ has developed a document that provides guidelines for professional training, considering that addressing violence against women in undergraduate courses can help students to be more comfortable in the future when dealing with these cases. The document highlights that students should develop appropriate skills, such as when and how to ask questions; the best way to respond to women; what emotional responses can affect care; how to adopt a non-blaming and non-judgmental stance; how to collect traces (when necessary); know the laws and basic services for care, among others^(22)^. The simulation proved to be in line with the WHO guidelines, as the students reported various skills developed through the practice, both procedural and attitudinal, such as a holistic approach, conflict mediation, active listening, and respect.

In the context of developing attitudinal skills, researchers analyzed the protocols available to guide professionals on IPV in the Spanish health system, conducting groups with practicing doctors to see how these instruments were used in practice. Professionals reported feelings of fear, frustration or helplessness as obstacles to dealing with the problem of IPV, and it is essential that there is training so that professionals’ emotional responses do not have a negative influence on caring for women^(25)^. In our study, we found that these feelings appear and can be managed from the time they are undergraduates, so it is essential to work on this issue with students.

The medicine and nursing students who took part in the simulation demonstrated the importance of welcoming and establishing a bond with the adolescents, identifying these as key attributes of working in PHC. The work process in PHC units in Brazil should prioritize the establishment of bonds and welcoming between teams and the population, which should be present in all healthcare relationships^(6),(26)^.

In this study, qualitative data was collected during the debriefing. Our findings demonstrated the importance and wealth of information, reflections and learning that this stage of the simulation provided for the students. This aspect is corroborated by a literature review that analyzed 140 studies, highlighting the importance of debriefing for teaching and learning in nursing education^(27)^. The relevance of holistic debriefing stands out, as it enables the acquisition of knowledge, skills and attitudes to be articulated in its formative and summative domains when dealing with a complex event.

Researchers from a German university implemented an elective course for medical students on domestic violence and highlighted in their findings the importance of the subject being covered in undergraduate classes since the participants had gaps in their knowledge^(17)^. These data corroborate those found in this study, highlighting once again the importance of this activity for the training of future professionals in a significant way.

The limitations of this study include the fact that it was a specific sample of students from a public university in the final years of their medical and nursing degrees. It did not include students from the private sector, other health courses, or other years of undergraduate study. Furthermore, it was not possible to carry out the simulations in an interprofessional manner due to differences in the schedules available between the students.

Despite its limitations, this study has shown how simulating IPV care for an adolescent in PHC is a powerful tool for the teaching-learning process in undergraduate health courses. The qualitative look at the process also allowed us to understand that this strategy can be coherent with complex themes that involve the acquisition and experimentation of cognitive, procedural, and attitudinal skills. We suggest that simulations be carried out with students from public and private universities and other undergraduate health courses, in an interprofessional manner.

## Conclusion

Revisiting the aim of this study, the use of clinical simulation has made relevant contributions to the teaching-learning process of undergraduate health students on the care of adolescent girls in a situation of IPV, in the context of PHC. There were reflections on the specificities of caring for adolescent girls in situations of IPV, such as the difference between notification and reporting and the autonomy of adolescents during care. It also fostered the development of new procedural and attitudinal skills, such as a holistic approach, conflict mediation, active listening, respect, and the importance of asking directly about the occurrence of violence. In this way, the attributes of PHC were mobilized and incorporated, enabling students to be better prepared for professional practice when they encounter these situations, contributing to better health care.
