# Supplementary material for: Clinical simulation on intimate partner violence in adolescent girls: contributions to health teaching
Source: Rev Lat Am Enfermagem. 2025 Feb 17;33:e4470. doi: 10.1590/1518-8345.7441.4470 (PMC11835003; doi:10.1590/1518-8345.7441.4470)
Supplement: Translation

# Translation: Simulação clínica sobre violência por parceiro íntimo em mulheres adolescentes: contribuições para o ensino-saúde*

**DOI:** 10.1590/1518-8345.7441.4471

**Authors:** Daniella Yamada Baragatti, Leticia da Silva Scotto, Claudia Adão Alves, Ana Paula de Miranda Araújo Soares, Fernanda Berchelli Girão, Diene Monique Carlos

**Affiliations:** 1 Universidade Federal de São Carlos, Departamento de Enfermagem, São Carlos, SP, Brasil; 2 Bolsista do Conselho Nacional de Desenvolvimento Científico e Tecnológico (CNPq), Brasil; 3 Universidade Federal de São Carlos, Departamento de Medicina, São Carlos, SP, Brasil; 4 Universidade Federal de São Carlos, São Carlos, SP, Brasil; 5 Bolsista da Coordenação de Aperfeiçoamento de Pessoal de Nível Superior (CAPES), Brasil; 6 Universidade de São Paulo, Escola de Enfermagem de Ribeirão Preto, Centro Colaborador da OPAS/OMS para o Desenvolvimento da Pesquisa em Enfermagem, Ribeirão Preto, SP, Brasil

**Keywords:** Violência por Parceiro Íntimo, Violência de Gênero, Violência Doméstica, Adolescente, Simulação de Paciente, Atenção Primária à Saúde

## Abstract

**Objetivo:**

conhecer as contribuições do uso de simulação clínica, com graduandos da área da saúde, para o atendimento à mulher adolescente em situação de violência por parceiro íntimo no contexto da Atenção Primária à Saúde.

**Método:**

estudo qualitativo, desenvolvido com 30 graduandos em Medicina e 28 em Enfermagem de uma universidade pública. A coleta de dados foi mediada pelo *debriefing* holístico, com posterior análise temática, reflexiva e indutiva.

**Resultados:**

emergiram dois temas: A complexidade da violência e Competências desenvolvidas com o uso da simulação. Os estudantes trouxeram a complexidade do atendimento devido a aspectos familiares, particularidades da população, ambivalência de sentimentos em um relacionamento violento e o limite entre a preservação da autonomia e a obrigatoriedade da denúncia. Foram desenvolvidas e exercitadas diversas competências, como habilidade de mediação de conflitos, respeito, vínculo, acolhimento e reconhecimento da rede de apoio.

**Conclusão:**

o uso da simulação se apresentou como potente ferramenta para o processo ensino-aprendizagem na graduação desses cursos, ao permitir reflexões sobre as especificidades no atendimento de adolescentes. O olhar qualitativo ao processo também permitiu aprofundar como esta estratégia pode ser coerente a temas complexos, que envolvem a aquisição e experimentação de competências cognitivas, procedimentais e atitudinais.

## Destaques

**(1)** A simulação se mostrou uma potente ferramenta para o ensino de cursos da saúde. **(2)** O olhar qualitativo permitiu aprofundar as contribuições em tema complexo. **(3)** Os estudantes desenvolveram e exercitaram competências para manejo de tema sensível. **(4)** Atributos da Atenção Primária à Saúde foram mobilizados no processo. **(5)** O *debriefing* holístico permitiu uma construção original e pertinente à temática.

## Introdução

A Violência por Parceiro Íntimo (VPI) é a forma mais comum de violência experimentada por mulheres globalmente. Trata-se de qualquer comportamento de parceiros íntimos, atuais ou anteriores, contra as mulheres que cause danos físico, psicológico ou sexual, incluindo violência física, abuso emocional, estupro, bem como comportamentos controladores e coercitivos^(1)^.

As adolescentes também podem estar expostas a esse tipo de violência, conhecida como *“Teen dating violence”* ou *“Dating violence”*. Este tipo de violência traz particularidades do adolescer, podendo ocorrer pessoalmente ou por meios eletrônicos, entre parceiros eventuais ou contínuos^(2)^. A violência entre parceiros íntimos adolescentes tem um profundo impacto na saúde, nas oportunidades e no bem-estar ao longo da vida, podendo começar cedo e durar a vida toda. As jovens acometidas por esse tipo de violência estão mais propensas à depressão, sintomas de ansiedade, uso de cigarros, álcool e outras drogas, envolvimento em atos infracionais, vivência de *bullying* e comportamentos suicidas^(3)^.

A VPI tem raízes nas questões de gênero, que são papéis sociais atribuídos às pessoas. As construções de gênero são desiguais, sendo que as instruções normativas sociais concedem poder ao masculino^(4)^, podendo resultar na naturalização da violência em uma relação íntima.

No mundo, 24% das jovens entre 15 e 19 anos relataram ter sofrido alguma violência física e/ou sexual por parceiro íntimo ao longo da vida, sendo que 16% relataram essa violência no último ano. No Brasil, 17% das jovens relataram as mesmas violências ao longo da vida e 10% no último ano^(5)^.

Em geral, o principal ponto de atenção para situações de VPI é a Atenção Primária à Saúde (APS). A APS é o primeiro nível de atenção em saúde e a principal porta de entrada para atendimento no Sistema Único de Saúde (SUS), devendo desenvolver uma atenção integral que impacte positivamente na situação de saúde das coletividades. É norteada por diversas políticas de saúde que evidenciam a importância do combate às situações de violência^(6)^. Para tal, todo o processo de formação profissional em saúde deve envolver estratégias de ensino que possibilitem a vivência e reflexões sobre a temática, o que deve ocorrer desde a graduação.

A simulação clínica é uma das estratégias de ensino que podem ser utilizadas para se trabalhar com estudantes e profissionais da área da saúde sobre VPI entre adolescentes. Baseada na experiência dos participantes, é uma tecnologia que utiliza uma ou mais estratégias para promover, melhorar ou validar competências. Entende-se por competência o processo de aquisição e construção de conhecimentos, habilidades e atitudes, num contexto social, cultural, histórico e político^(7)^.

Uma revisão sistemática encontrou que a simulação clínica foi identificada como um método educacional eficaz para uma aprendizagem autêntica, na qual os estudantes de graduação da saúde cultivaram seu conhecimento e motivação para aprender^(8)^. Apesar desta conclusão, revisão de literatura recente evidenciou que a maioria dos cenários de simulação validados no Brasil versam sobre temas como urgência e emergência, cuidado materno e estomaterapia, justificando o olhar a fenômenos complexos como a violência^(9)^. Embora prevalentes na prática clínica, são pouco abordados na formação, mesmo com metodologias tradicionais^(2)^.

Uma das etapas de uma simulação clínica é o *debriefing,* que ocorre logo após a experiência desta estratégia. Buscando promover a aprendizagem reflexiva na simulação, pesquisadoras desenvolveram um guia holístico de *debriefing* que incluiu aspectos formativos e sumativos de orientação para auxiliar os educadores na condução desta importante etapa^(10)^.

Assim, considerando a relevância de profissionais enfermeiros e médicos nos serviços de saúde e a importância de o tema ser trabalhado na graduação, este estudo visou conhecer as contribuições do uso de simulação clínica com graduandos da área da saúde, para o atendimento à mulher adolescente em situação de violência por parceiro íntimo no contexto da Atenção Primária à Saúde. Entende-se que este olhar se coaduna com as perspectivas de fortalecimento do trabalho de Enfermagem e Saúde para atender às necessidades de adolescentes, em especial no contexto da APS por meio de ações sensíveis culturalmente e ao gênero. Ademais, pode trazer uma ferramenta para instrumentalização de docentes e enfermeiros no cuidado à saúde desta população^(11)^.

## Método

### Tipo ou delineamento do estudo

Estudo descritivo-exploratório, com abordagem qualitativa^(12)^, que seguiu o referencial conceitual do *debriefing* holístico^(10)^. Os aspectos contidos no *Consolidated Criteria for Reporting Qualitative Research* (COREQ) nortearam o desenho e a apresentação desta investigação.

### Local e cenário do estudo

O local de estudo foi uma universidade pública localizada no interior do estado de São Paulo, que possui 254.857 habitantes, em uma área territorial de 1.137 km^2^. A universidade possui 64 cursos de graduação e 15.518 estudantes estavam matriculados em 2023. A escolha da amostra foi por conveniência das pesquisadoras (já terem contato com os docentes e estudantes) e pela universidade utilizar estratégias de simulação na graduação.

### Período

O período da coleta de dados ocorreu entre agosto e outubro de 2023.

### Critérios de seleção e participantes

Os critérios de inclusão para os participantes foram: ser estudante de graduação de Medicina e Enfermagem da universidade do estudo; ser maior de 18 anos; ter tido contato com disciplinas teóricas que tratavam do da violência ou do cuidado à criança, adolescente e mulher; comparecer no dia e horários combinados para a atividade de simulação e participar de todas as etapas propostas. Já o critério de exclusão foi estar afastado das atividades universitárias por quaisquer motivos.

Para a seleção dos participantes, a pesquisa contou com apoio para divulgação da Coordenadoria de Comunicação Social da Universidade por meio de *e-mails* disparados às coordenações dos cursos, assim como pela publicação em meio eletrônico nas plataformas e redes sociais dos Centros Acadêmicos da Universidade. Não houve contato com as pesquisadoras nesta fase.

Participaram da atividade aqueles que entraram em contato com a pesquisadora e compareceram nos dias agendados para a realização da simulação, na Unidade de Simulação em Saúde, que possui ambientes simulados de alta similitude a consultórios de APS. Todos os estudantes que compareceram aceitaram participar da pesquisa.

### Coleta de dados

A simulação clínica ocorreu norteada pelo cenário “Violência por parceiro íntimo contra a mulher adolescente na Atenção Primária à Saúde”, fundamentado na Taxonomia de Bloom para construção dos objetivos dos processos educacionais baseados nos domínios cognitivos, procedimentais e atitudinais; nas normas de práticas recomendadas para o *design* de simulação pela *International Nursing Association for Clinical Simulation and Learning* ^(13)^; e roteiro para construção de cenário simulado^(14)^. Para a finalização do cenário, foi utilizado o guia “Três estágios do *debriefing* holístico”^(10)^. Ressalta-se que o cenário foi elaborado e validado por 26 juízes especialistas na temática de simulação clínica e violência.

As simulações com estudantes de Medicina e Enfermagem aconteceram separadamente e o objetivo geral de aprendizagem do cenário foi que o estudante tivesse, ao final da atividade, a capacidade de desenvolver o cuidado da mulher adolescente em situação de VPI.

O pré-*briefing* ocorreu por meio do envio prévio de uma cartilha informativa sobre violência contra mulheres^(15)^ e um infográfico sobre VPI entre adolescentes^(3)^. O *briefing,* com duração entre dez a quinze minutos*,* iniciou-se com a apresentação do ambiente, equipamentos, recursos, apresentação das facilitadoras, informações aos estudantes quanto ao objetivo de aprendizagem a ser atingido no cenário simulado, além do contrato de trabalho, como respeito mútuo, confidencialidade e ética. Os estudantes receberam o Termo de Consentimento Livre e Esclarecido (TCLE), responderam a um questionário sobre seu perfil socioeconômico e receberam também a descrição do caso clínico, lida conjuntamente pelo grupo. Os estudantes foram informados, no dia da simulação clínica, sobre a formação das pesquisadoras e a razão para o desenvolvimento do trabalho. Ademais, caso não concordassem em participar da pesquisa, ainda assim poderiam participar da simulação.

Na sequência, foi definida uma dupla de estudantes, que atenderia a adolescente em situação de violência e sua mãe, para participarem do cenário, com tempo de execução máxima de trinta minutos. Os demais estudantes permaneceram como espectadores do cenário em um espaço separado, na própria unidade de simulação.

Os cenários foram gerenciados por duas facilitadoras, mulheres cisgênero, enfermeiras doutoras e pesquisadoras de uma universidade pública, sendo uma pós-doutoranda e outra docente. Ambas, expertas na temática, dispunham de um fluxograma para auxiliar na condução das atividades, buscando atingir os objetivos de aprendizagem inicialmente propostos. Não houve a participação de outras pessoas no cenário de pesquisa além das descritas —pesquisadoras facilitadoras, estudantes e atrizes.

O *debriefing* foi gravado, utilizando-se um aplicativo de gravação de voz para aparelhos celulares e transcrito manualmente. A discussão foi conduzida por meio de um instrumento elaborado pelas pesquisadoras, baseado nos “Três estágios do *debriefing* holístico”^(10)^, a saber: (1) Focado (Autorreflexão imediata dos estudantes após a experiência prática), com as seguintes questões: “Como você se sente em relação à experiência de simulação? O que você aprendeu ao participar da experiência de simulação? Quais habilidades você acha que desenvolveu durante a experiência de simulação?”; (2) Formativo (*debriefing* contínuo com o grupo maior durante toda a simulação) com as questões: “Como um grupo, como vocês se sentiram durante a simulação? Como um grupo, o que vocês acham que sabem/entendem melhor agora? Como um grupo, vocês acham que esta experiência e a discussão em simulação ajudaram a desenvolver habilidades psicomotoras? Por que ou por que não? O que aprenderam durante esta simulação que é semelhante ao que aprenderam no curso de graduação? O que é diferente?”; e (3) Sumativo (reflexão final com o grupo maior e sua aplicação ao aprendizado obtido), com as questões: “Como vocês se sentiram após a experiência de simulação? Como vocês perceberam a progressão no aprendizado por meio desta experiência? Que tipo de habilidades vocês acham que desenvolveram por meio da experiência de simulação? Vocês acreditam que a experiência e os conceitos de violência contra adolescentes que discutimos no pré-*briefing* estão claros para vocês, com base nas evidências de melhores práticas? Justifique sua resposta. Vocês acham que a atividade de simulação contribuiu para sua atuação profissional ao lidar com adolescentes em situação de violência por parceiro íntimo?”.

Foi realizado um primeiro teste-piloto para verificar a adequação do instrumento de coleta de dados, não sendo necessárias mudanças; os dados do teste-piloto foram incorporados à pesquisa. Não houve repetições dos grupos e a saturação de dados, ou seja, o aprofundamento dos elementos que respondiam às questões de pesquisa foi alcançado no quinto grupo focal, sendo realizados mais dois já agendados. Imediatamente após a simulação clínica, a pesquisadora principal realizou anotações sobre as simulações em um diário de campo, que auxiliou na discussão dos achados.

No total, foram sete simulações com grupos de aproximadamente oito estudantes, com a participação de 28 graduandos em Enfermagem e 30 em Medicina. Contabilizando todas as etapas, a atividade com menor duração foi de 2h35min e a com maior duração, 2h55min.

Para a apresentação dos dados, os participantes foram identificados conforme o curso a que pertenciam, sendo Med para Medicina e Enf para Enfermagem, seguidas de um número sequencial, segundo a ordem em que falaram durante a atividade.

### Tratamento e análise dos dados

Os dados do perfil dos participantes, coletados por meio do questionário, foram apresentados por meio de estatística descritiva. Os dados do *debriefing* foram transcritos na íntegra, utilizando o aplicativo *Documentos Google*, e analisados a partir da técnica de análise temática reflexiva e indutiva, ou seja, emergindo a partir dos dados^(16)^. Foram gerados 16 códigos iniciais e 5 intermediários, (“A complexidade da violência”, “Sentimentos e emoções envolvidos no atendimento”, “Importância do acolhimento e vínculo”, “As especificidades no cuidado de casos de violência contra adolescentes” e “Habilidades desenvolvidas com o uso da simulação”), e que, por sua vez, foram agrupados em dois temas finais.

Para maior confiabilidade deste processo, foram seguidos os seguintes critérios: (1) utilização de diário de campo que permitiu cuidadoso e completo registro do processo de coleta e inferências em campo; (2) construção dos códigos e temas entre duas pesquisadoras (1ª e 2ª autora), consensualizado por uma terceira (última autora); (3) checagem dos dados interpretados junto aos participantes para verificação de sentido ou necessidade de novas inserções — 17 devolutivas por *e-mail* foram realizadas, não sendo incorporadas novas informações.

### Aspectos éticos

A pesquisa foi aprovada pelo Comitê de Ética em Pesquisa da Universidade Federal de São Carlos, sob CAAE 63438122.1.0000.5504.

## Resultados

Dentre os 58 estudantes, a maioria possuía entre 20 e 25 anos (medicina n=25, 83%; enfermagem n=21, 75%). Quanto à cor, a maioria se declarou branca (medicina n=22, 73%; enfermagem n=19, 68%). Todos eram cisgêneros, sendo que da Medicina 17 se declararam como homens cis (57%) e 13 (43%) como mulheres cis. Já entre os estudantes de Enfermagem, três (11%) se declararam homens cis e 25 (89%) como mulheres cis.

Após análise dos dados qualitativos, os resultados foram divididos em dois temas finais: “A complexidade da violência” e “Competências desenvolvidas com o uso da simulação”.

### A complexidade da violência

Este tema abordou a complexidade de elementos que envolve o atendimento a casos de VPI contra adolescentes mulheres. Um primeiro aspecto se relacionou à apresentação da situação — engloba aspectos familiares mais amplos que necessitam ser compreendidos para um atendimento integral. A presença da mãe no atendimento inicialmente, bem como conflitos familiares, incluindo suspeita de violência doméstica, tornaram a abordagem mais desafiadora:

*E explorar também a questão de o pai beber, que é talvez uma questão de um alcoólatra no ambiente familiar, então daria para buscar um pouco mais essa informação também”*. (Med 28)

*Depois eu teria o interesse também de conversar só com a mãe, tem toda a questão familiar, do ambiente familiar, porque ela foge desse ambiente. Não seria legal falar isso na frente dela.* (Enf 5)

*São duas pessoas ativas no atendimento, com histórias diferentes. Então às vezes quando a gente atende, é uma pessoa só ou a outra pessoa, quando são duas, está ali complementando a história da outra. Essa é a primeira vez que a gente pegou duas pessoas que tinham visão diferente dentro do mesmo assunto.* (Med 21)

*Eu achei muito complexo, por que tem a questão do pai, eu não sabia se podia perguntar “seu pai te bateu? Rolou outras violências?” Ou se ela presencia outras violências com a mãe (...) tem a questão do namorado, são muitas questões complexas em um ser só.* (Enf 1)

Conforme evidenciado na última fala, desvelaram-se particularidades da vivência da VPI na adolescência. Os participantes trouxeram maiores dificuldades na abordagem e desenrolar do atendimento, visto a adolescente ter procurado a unidade não por uma necessidade explícita.

É uma adolescente e ela é meio fechada. (Med 5)

*Ela não queria estar aqui, ela não queria estar na consulta, ela veio forçada pela mãe, então ela já estava desinteressada em tudo. Ela estava aqui por que a mãe obrigou*. (Enf 8)

A ambivalência na relação, o estar apaixonada e uma vivência de violência mais velada — dada pelo ciúme e controle — emergiram nos relatos como aspectos desafiadores.

*Ela também não se via em situação de violência né. Então, isso foi bem mais desafiador*. (Enf 17)

*Porque a gente tem que lembrar da questão de maturidade mesmo, de desenvolvimento. Então a idade dela, ela ainda não tem a maturidade de desenvolvimento suficiente para entender completamente e fazer o julgamento do que é certo e errado.* (Med 4)

*No começo ela fala que sentia falta de sair com as amigas, que o namorado proibia e chantageava, e aí quando a gente pergunta de rede de apoio ela fala que só tem o namorado e o restante é tudo falso. (*Enf 15*)*

*E pelo visto, é uma pessoa muito fechada, até a posição que ela sentou e tal. Então é muito difícil você chegar com uma ideia, falando uma coisa, sendo que ela confia muito no namorado né, até por que ela não consegue lidar com os próprios pais*. (Med 6)

Vislumbra-se pela última fala que os participantes tiveram dificuldades em construir o diálogo com um perfil adolescente, que muitas vezes num primeiro contato é verbalmente menos elaborado. O não contato com o público adolescente ao longo do percurso acadêmico em serviços de saúde trouxe maiores desafios para estabelecimento da relação:

É muito mais difícil, eu acho, a gente estabelecer vínculo com a adolescente, enfim, que às vezes só tem mais contato com os pais, com as amizades, né. (Enf 19)

*Eu achei um pouco mais complicado de lidar, por que agora a gente está lidando com uma adolescente, coisa que a gente nunca lidou.* (Med 12)

A questão de “ser menor de idade” foi recorrente na discussão da situação experienciada, em especial por dúvidas sobre a denúncia legal, manutenção do sigilo e vínculo, relações de poder estabelecidas entre a adolescente e o adulto e melhor manejo do caso:

*Eu fiquei com muita dúvida nisso, de como falar que não era normal um relacionamento de uma criança de 13 anos com um adulto de vinte e poucos anos. Eu não saberia como falar isso. Não é crime?* (Med 2)

*No caso seria só com o conselho ou teria um outro órgão para acionar também? Talvez escola? A delegacia da mulher, no caso, se comunica como com essa rede?* (Med 6)

*Essa questão dos ciúmes né, a diferença na relação de poder entre um adulto de 21 anos e uma adolescente de 13. E até tem mais umas coisas que eu coloquei e se ela tem medo de ir contra o namorado, por que eu queria mais explorar esse balanço de poder.* (Enf 17)

*Ir conversando mais com a paciente, fazer ela entender que isso é problemático, não falar da denúncia logo de cara, se não, ela nunca mais volta.* (Enf 7)

### Competências desenvolvidas com o uso da simulação

Neste tema, emergiram as competências que os estudantes adquiriram ou exercitaram por meio da simulação em termos de conhecimentos, habilidades e atitudes. Pela particularidade do objetivo da simulação, é importante destacar que tratar da violência, em especial contra adolescentes, é visto como um “*tema tenso, pesado* (Enf 12)”, que traz sentimentos que precisam ser falados e manejados:

*Eu senti um grande desconforto para ser sincera. Porque uma situação muito comum, que eu lembrei de mães amigas minhas, que eu vi o jeito que elas conversavam e tudo mais, o que elas passam, como se sentem, então traz aquele desconforto e aquelas lembranças.* (Enf 23)

*Então, me dá angústia, vivenciar um caso de violência, ainda mais com o caso dela sendo uma adolescente menor de idade.* (Med 3)

*Eu acho que é muito difícil, eu até me recusei a fazer a simulação por não me sentir confortável. E não é uma questão de entender e aceitar, eu não me sinto bem fazendo isso. Então eu prefiro observar, ver como as pessoas fazem para tentar aprender e tirar algo dali*. (Med 7)

A última fala demonstra a coerência do uso da estratégia de simulação para facilitar o processo ensino-aprendizagem, tornando o tema mais viável e palatável. Neste sentido, os participantes relataram mobilizar conhecimentos já adquiridos, como a importância do acolhimento, vínculo e continuidade do cuidado, atributos essenciais da APS, bem como a articulação a aspectos teóricos da violência e rede de apoio:

*Eu acho que pela primeira consulta a gente tinha que acolher, mostrar que é um ambiente seguro e que a gente estava ali para ouvir sem julgamento*. (Enf 2)

*Ela fez a criação do vínculo com os pacientes, de modo que não acusasse ninguém, nem fazendo ninguém se sentir mal, para ter essa continuidade do cuidado. Acho que a pior coisa na APS é isso, você espantar o paciente no primeiro atendimento e ele não voltar mais.* (Med 14)

*Uma coisa que, eu acabei voltando, é que quando a gente estava estudando sobre violência familiar e doméstica, as pessoas que conviveram em uma casa em que há violência, tem uma maior tendência a perpetuar esse cenário violento. E é o que acabou aparecendo no caso, que a mãe veio de uma família que tinha violência, ela vive em um cenário de violência, e aparentemente a filha também está entrando em um cenário, também de violência. Então eu acredito que, explorar mais, como a filha vê o próprio caso da mãe pode ajudar a refletir em como que ela também está entrando em uma situação, talvez parecida, e pode encaminhar para o mesmo futuro*. (Enf 6)

*Saber se ela tem amigas, algum outro familiar de quem ela é mais próxima, para tentar ver a rede de apoio. Com mais tempo, talvez como uma forma de vínculo, encaminhar para um psicólogo da unidade, grupo de terapia. Por que ela não tem amigos na escola? Que que aconteceu, se aconteceu alguma coisa, se o namorado a afasta dessas amizades.* (Med 15)

Neste sentido, os estudantes puderam exercitar o cuidado em ato na VPI entre adolescentes na APS; aspectos desvelados para esta habilidade foram o olhar integral, para além de sinais e sintomas presentes, a escuta ativa e a mediação de conflitos. Aspectos atitudinais, sem “receita prévia”, como respeito e tom de voz, foram citados:

*Acho que tem outro ponto também que eu havia esquecido de falar que é a escuta ativa. Então a adolescente falando, você prestando atenção naquilo que ela está falando, olhando para ela (...). Eu já tinha lido em alguns livros justamente que o fato de você ficar quieto um tempinho a mais, faz com que a pessoa fale alguma nova informação que talvez ela não falaria se você interrompesse com uma pergunta ali naquele momento*. (Med 9)

*Bom, eu acho que assim, é uma coisa bem desafiadora, por que às vezes a gente se preocupa com outras coisas que a gente tenta identificar e acaba passando despercebido esses sinais e se tem alguma coisa extra acontecendo*. (Enf 1)

*O que eu percebi é que assim, eu preciso aprender a maneira como abordar esse caso, o jeito que fala, o tom de voz, eu ainda tenho um pouquinho de dificuldade em conduzir esses casos.* (Enf 2)

Com a possibilidade de simular um atendimento, o cuidado na VPI contra adolescentes mulheres na APS foi compreendido como um processo que demanda tecnologias leves, tempo e ações ampliadas. Este elemento facilitou a problematização de discussões sobre o processo de denúncia, bem como o equilíbrio necessário nestas situações:

É uma situação complicada né, você não pode falar para ela tipo: “olha termina com seu namorado, por que...”, você não pode falar isso. Acho que a forma mais segura de fazer isso, seria ela entender as repercussões desse relacionamento na vida dela e, para isso seria um processo, não seria em uma consulta, seria um bom tempo de conversa, tanto com os profissionais de saúde, quanto com a mãe e o pai teria que ter essa conscientização. (Med 4)

*Essa é uma habilidade que eu tenho que desenvolver, por que eu quero saber tudo da paciente para ontem. Já penso “vamos sair disso mulher, você está sendo oprimida”.* (Enf 12)

Finalmente, os participantes trouxeram a relevância da simulação, colocando-os como pessoas ativas na construção do processo ensino-aprendizagem. O *debriefing* foi apontado como etapa importante, para elaboração do sentido e apreendido, bem como da aquisição de novas competências por meio de uma aprendizagem significativa:

*Eu acho legal da simulação, porque, a gente vê a teoria, os sinais, e vocês facilitam para a gente no sentido de colocar quase todos. Então, a pessoa evita falar, ela não olha para o seu rosto para responder, o conflito que pode acontecer entre a mãe e a menina, enfim. Isso é bom porque acentua para a gente que aquilo vai acontecer, a gente não só lê o que está na teoria.* (Enf 16)

*Então, eu acredito que a simulação tenha contribuído muito para a gente entender o adolescente, as angústias, as demandas, e tudo mais.* (Med 27)

*Eu achei legal também a questão de a gente tirar dúvidas, porque como a gente tinha comentado, essa questão da notificação compulsória ou da denúncia, deixar isso registrado, isso a gente não tinha visto tão a fundo, achei algo interessante.* (Med 7)

## Discussão

Os estudantes perceberam a complexidade do atendimento de casos de VPI contra mulheres adolescentes no contexto da APS devido a: aspectos familiares amplos; a presença da mãe durante o processo; as particularidades da VPI na adolescência; a dificuldade de diálogo neste período; a percepção da ambivalência de sentimentos em um relacionamento violento e o equilíbrio entre a preservação da autonomia da pessoa adolescente e a obrigatoriedade da denúncia do caso. Apesar dessa complexidade, de terem que lidar com sentimentos angustiantes despertados pelo fenômeno, os estudantes desenvolveram diversas competências, como a habilidade de mediação de conflitos e atitudes como o respeito, identificando que o cuidado na VPI se estabelece como um processo. Para desenvolvê-lo, foram mobilizados atributos da APS, como a necessidade de continuidade do cuidado por meio do vínculo, acolhimento e reconhecimento da rede de apoio.

O perfil sociodemográfico dos participantes corrobora estudo na área^(17)^, em que a maioria dos graduandos na área da saúde tem idade até 25 anos, é branca e se declara como mulher cisgênero. O cuidado a adolescentes em situação de violência tem muitas especificidades^(3)^. Foram recorrentes, durante a simulação, dúvidas sobre a diferença entre notificação e denúncia e o limite da autonomia do adolescente. Todos os casos de violência são de notificação compulsória — trata-se de uma comunicação obrigatória à autoridade de saúde, realizada pelos profissionais, a partir da ocorrência de suspeita ou confirmação de agravos específicos, permitindo a análise dos dados e podendo fornecer subsídios para as tomadas de decisão. Tal aspecto difere da denúncia e mesmo da articulação do cuidado da pessoa adolescente, por vezes negligenciado^(18)^. Considerar o cuidado das adolescentes para além de prismas convencionais da saúde (a partir de fatores de risco e proteção), e incluí-las como pessoas ativas socialmente, em especial grupos vulneráveis, é colocado como força motriz para a participação significativa na ação global acelerada para a saúde desta população^(19)^.

No Brasil, conforme a Lei nº 8069 que dispõe sobre o Estatuto da Criança e do Adolescente (ECA), o adolescente é sujeito de direitos que pode ser atendido em serviço de saúde de forma autônoma^(20)^. Os estudantes, em respeito a isso, consultaram a adolescente sobre o desejo ou não de que a mãe se retirasse para ouvi-la sozinha durante a simulação. Ademais, embora no ECA não haja nada específico sobre proibição de namoro entre um adolescente e um adulto, o Artigo 217-A do Código Penal brasileiro considera crime a conjunção carnal ou ato libidinoso com menor de 14 anos, sendo prevista pena de reclusão de oito a 15 anos^(21)^. Segundo o ECA, é obrigatória a denúncia ao Conselho Tutelar em casos de violências suspeitas ou confirmadas contra crianças e adolescentes. Embora haja a obrigatoriedade da denúncia por poder estar acontecendo um crime, durante o cuidado da APS é preciso refletir sobre este processo, inclusive com a adolescente, além de estabelecer cuidados ampliados para ela. É preciso entender a dinâmica familiar, trazendo a adolescente como centro deste cuidado em consonância ao ECA e às demais legislações^(20-22)^.

Ainda em relação ao atendimento desenvolvido na simulação, os participantes relataram dúvidas em como abordar a adolescente e se poderiam questionar diretamente sobre violências. Pesquisadores de uma Universidade americana elaboraram um guia para auxiliar estudantes de enfermagem a reconhecerem e auxiliarem adolescentes que estejam sofrendo violência no namoro. Destacaram a importância de perguntar diretamente sobre este fenômeno, pois muitos adolescentes têm medo de contar esses problemas ou podem não perceber os comportamentos do parceiro como violentos^(23)^.

Um estudo analisou os currículos de graduação em enfermagem em instituições públicas no Brasil em relação à abordagem da violência contra as mulheres^(24)^. Foi identificado que a abordagem à saúde do adolescente refere-se mais aos aspectos de violação de direitos sexuais e reprodutivos, com a preocupação relacionada à gravidez na adolescência^(24)^. Este achado reforça a importância da simulação sobre violência contra uma mulher adolescente para os graduandos, uma vez que trata-se de uma abordagem que vai além dos aspectos biológicos relacionados às violências e que é pouco desenvolvida nos currículos de graduação. Tal aspecto se coaduna ainda com a importância de tal temática para a prevenção de violências baseadas no gênero futuras.

Reconhecendo a importância de preparar os estudantes, principalmente de medicina, enfermagem e obstetrícia, para cuidarem de mulheres em situação de violência, a Organização Mundial de Saúde (OMS)^(22)^ desenvolveu um documento que traz diretrizes para a formação profissional. Considera-se que a abordagem sobre violência contra as mulheres na graduação pode auxiliar os estudantes a ficarem mais confortáveis no futuro ao lidar com esses casos. Destaca que os estudantes devem desenvolver competências adequadas, como quando e como perguntar; a melhor forma de responder às mulheres; quais as respostas emocionais que podem afetar o atendimento; como adotar uma postura que não culpe e não julgue; saber como recolher vestígios (quando necessário); conhecer as leis e serviços básicos para atendimento, dentre outras^(22)^. A simulação demonstrou estar em consonância com as diretrizes da OMS, pois os estudantes relataram diversas competências desenvolvidas com a prática, tanto procedimentais quanto atitudinais, como o olhar integral, a mediação de conflitos, a escuta ativa e o respeito.

No âmbito do desenvolvimento de competências atitudinais, pesquisadores analisaram os protocolos disponíveis para orientar os profissionais sobre VPI no sistema de saúde da Espanha, realizando grupos com médicos atuantes para verificar como se dava a utilização desses instrumentos na prática. Os profissionais relataram sentimentos de medo, frustração ou impotência como obstáculos para enfrentar o problema da VPI, sendo fundamental que haja treinamento para que as respostas emocionais dos profissionais não tenham influência negativa no cuidado às mulheres^(25)^. Nesta pesquisa, encontrou-se que esses sentimentos aparecem e seu manejo pode ser exercitado desde a graduação, sendo, portanto, fundamental que essa temática seja trabalhada com os estudantes.

Os estudantes de Medicina e Enfermagem que participaram da simulação demonstraram a importância do acolhimento e do estabelecimento de vínculo com a adolescente, identificando serem esses atributos fundamentais do trabalho na APS. O processo de trabalho nas unidades de APS no Brasil deve priorizar o estabelecimento de vínculo e o acolhimento entre as equipes e a população, que devem estar presentes em todas as relações de cuidado^(26)^.

Nesta pesquisa, os dados qualitativos foram coletados durante o *debriefing.* Os achados demonstraram a importância e a riqueza de informações, reflexões e aprendizagens que esta etapa da simulação proporcionou aos estudantes. Tal aspecto é corroborado por uma revisão de literatura que analisou 140 estudos, destacando a importância do *debriefing* para o ensino e a aprendizagem da educação em enfermagem^(27)^. Destaca-se a pertinência do *debriefing* holístico, que permitiu articular a aquisição de conhecimentos, habilidades e atitudes nos seus domínios formativo e sumativo ante o manejo de fenômeno complexo.

Pesquisadores de uma universidade alemã implementaram um curso eletivo para estudantes de medicina sobre violência doméstica e destacaram, em seus achados, a importância da temática ser trabalhada em cursos de graduação, uma vez que os participantes apresentaram lacunas em relação a este conhecimento^(17)^. Estes dados corroboram aqueles encontrados pelo presente estudo, destacando novamente a importância significativa dessa atividade para a formação dos futuros profissionais.

Como limitações deste estudo destacamos o fato de se tratar de uma amostra específica de estudantes de uma universidade pública dos últimos anos de graduação em medicina e enfermagem. O mesmo não abrangeu estudantes da rede privada, de outros cursos da saúde ou de outros anos de graduação. Ademais, não foi possível realizar as simulações interprofissionalmente devido às diferenças de horários disponíveis entre os estudantes.

Apesar das limitações, este estudo demonstrou como a simulação de um atendimento de VPI contra uma adolescente na APS é uma potente ferramenta para o processo ensino-aprendizagem na graduação de cursos da saúde. O olhar qualitativo ao processo também permitiu compreender que esta estratégia pode ser coerente a temas complexos e que envolvem a aquisição e experimentação de competências cognitivas, procedimentais e atitudinais. Sugerimos que sejam realizadas simulações com estudantes de universidades públicas e privadas e de outras áreas de graduação na saúde, de maneira interprofissional.

## Conclusão

Retomando o objetivo deste estudo, o uso da simulação clínica trouxe contribuições relevantes para o processo ensino-aprendizagem de graduandos da saúde sobre o atendimento da mulher adolescente em situação de VPI, no contexto da APS. Houve reflexões sobre as especificidades no atendimento de adolescentes mulheres em situação de VPI, como a diferença entre notificação e denúncia e a autonomia das adolescentes durante os atendimentos. Ademais, favoreceu o desenvolvimento de novas competências procedimentais e atitudinais, como o olhar integral, a mediação de conflitos, a escuta ativa, o respeito e a importância de se perguntar diretamente sobre a ocorrência de violência. Assim, os atributos da APS foram mobilizados e incorporados, possibilitando que estudantes estejam melhor preparados para a prática profissional quando se depararem com essas situações, contribuindo para um melhor atendimento.

# Translation: Simulación clínica sobre violencia de pareja en mujeres adolescentes: aportaciones a la enseñanza en salud*

**DOI:** 10.1590/1518-8345.7441.4469

**Authors:** Daniella Yamada Baragatti, Leticia da Silva Scotto, Claudia Adão Alves, Ana Paula de Miranda Araújo Soares, Fernanda Berchelli Girão, Diene Monique Carlos

**Affiliations:** 1 Universidade Federal de São Carlos, Departamento de Enfermagem, São Carlos, SP, Brasil; 2 Becaria del Conselho Nacional de Desenvolvimento Científico e Tecnológico (CNPq), Brasil; 3 Universidade Federal de São Carlos, Departamento de Medicina, São Carlos, SP, Brasil; 4 Universidade Federal de São Carlos, São Carlos, SP, Brasil; 5 Becaria de la Coordenação de Aperfeiçoamento de Pessoal de Nível Superior (CAPES), Brasil; 6 Universidade de São Paulo, Escola de Enfermagem de Ribeirão Preto, Centro Colaborador de la OPS/OMS para el Desarrollo de la Investigación en Enfermería, Ribeirão Preto, SP, Brasil

**Keywords:** Violencia de Pareja, Violencia de Género, Violencia Doméstica, Adolescente, Simulación de Paciente, Atención Primaria de Salud

## Abstract

conocer las contribuciones del uso de la simulación clínica en la atención a mujeres adolescentes en situación de violencia de pareja en el contexto de la Atención Primaria de Salud.

estudio cualitativo con 30 estudiantes de pregrado de medicina y 28 de enfermería de una universidad pública de Brasil. La recogida de datos fue mediada por un *debriefing* holístico, con posterior análisis temático reflexivo e inductivo.

surgieron dos temas: la complejidad de la violencia y las competencias desarrolladas mediante el uso de la simulación. Los estudiantes plantearon la complejidad de la atención debido a los aspectos familiares, las particularidades de la población, la ambivalencia de sentimientos en una relación violenta y el límite entre preservar la autonomía y la obligación de denunciar. Se desarrollaron y practicaron diversas habilidades, como las de mediación de conflictos, respeto, vinculación, acogida y reconocimiento de la red de apoyo.

el uso de la simulación demostró ser una herramienta poderosa para el proceso de enseñanza-aprendizaje en cursos de pregrado, ya que permitió reflexionar sobre las especificidades de la atención a adolescentes. El abordaje cualitativo del proceso también permitió profundizar en cómo esta estrategia puede ser coherente con temas complejos que involucran la adquisición y experimentación de competencias cognitivas, procedimentales y actitudinales.

## Destacados

**(1)** La simulación demostró ser una poderosa herramienta para la enseñanza de cursos de salud. **(2)** El abordaje cualitativo permitió profundizar los aportes a un tema complejo. **(3)** Los estudiantes desarrollaron y ejercitaron competencias para el manejo de un tema sensible. **(4)** Los atributos de la Atención Primaria de Salud se movilizaron en el proceso. **(5)** El *debriefing* holístico permitió una construcción original y pertinente del tema.

## Introducción

La violencia de pareja (VP) es la forma más común de violencia que sufren las mujeres en todo el mundo. Se trata de cualquier comportamiento de la pareja actual o anterior contra la mujer que cause daño físico, psicológico o sexual, incluida la violencia física, el abuso emocional, la violación, así como el comportamiento controlador y coercitivo^(1)^.

Las adolescentes también pueden estar expuestas a este tipo de violencia, conocida como “*Teen dating violence*” o “*Dating violence*”. Este tipo de violencia es propio de la adolescencia y puede producirse en persona o por medios electrónicos, entre parejas ocasionales o continuas^(2)^. La violencia entre parejas adolescentes tiene un profundo impacto en la salud, las oportunidades y el bienestar a lo largo de la vida, y puede comenzar a una edad temprana y durar toda la vida. Las jóvenes afectadas por este tipo de violencia son más propensas a la depresión, los síntomas de ansiedad, el consumo de cigarrillos, alcohol y otras drogas, la participación en delitos, el acoso escolar y el comportamiento suicida^(3)^.

La VP tiene sus raíces en cuestiones de género, que son roles sociales asignados a las personas. Las construcciones de género son desiguales, y las instrucciones normativas sociales otorgan el poder al varón^(4)^, lo que puede dar lugar a la naturalización de la violencia en una relación íntima.

En todo el mundo, el 24% de las mujeres jóvenes de entre 15 y 19 años declararon haber sufrido violencia física y/o sexual por parte de un compañero íntimo a lo largo de su vida, y el 16% declararon haber sufrido esta violencia en el último año. En Brasil, el 17% de las jóvenes declararon haber sufrido la misma violencia a lo largo de su vida y el 10% en el último año^(5)^.

En general, el principal punto de atención para situaciones de VP es la Atención Primaria de Salud (APS). La APS es el primer nivel de atención de salud y la principal puerta de entrada a la atención en el Sistema Único de Salud de Brasil (SUS), que debe proporcionar una atención integral con un impacto positivo en la situación de salud de las comunidades y está orientada por diversas políticas de salud que enfatizan la importancia de combatir las situaciones de violencia^(6)^. Para ello, todo el proceso de formación profesional en salud debe involucrar estrategias de enseñanza que permitan vivenciar y reflexionar sobre el tema, lo que debe ocurrir desde los cursos de pregrado.

La simulación clínica es una de las estrategias de enseñanza que pueden ser utilizadas para trabajar con estudiantes y profesionales de salud sobre la VP en adolescentes. De acuerdo con la experiencia de los participantes, es una metodología que utiliza una o más estrategias para promover, mejorar o validar competencias. La competencia se entiende como el proceso de adquisición y construcción de conocimientos, habilidades y actitudes en un contexto social, cultural, histórico y político^(7)^.

Una revisión sistemática encontró que la simulación clínica fue identificada como un método educativo eficaz para el aprendizaje auténtico, en el cual los estudiantes de pregrado de salud cultivaron sus conocimientos y motivación para aprender^(8)^. A pesar de esa conclusión, una revisión bibliográfica reciente mostró que la mayoría de los escenarios de simulación validados en Brasil abordan temas como urgencia y emergencia, atención materna y estomaterapia, lo que justifica el abordaje de eventos complejos como la violencia^(9)^. A pesar de ser frecuentes en la práctica clínica, raramente son abordados en la formación, incluso con metodologías tradicionales^(2)^.

Una de las etapas de una simulación clínica es el *debriefing*, que tiene lugar inmediatamente después de la experiencia de esta estrategia. Con el fin de promover el aprendizaje reflexivo en la simulación, los investigadores desarrollaron una guía holística de *debriefing* que incluía aspectos formativos y sumativos para ayudar a los educadores a llevar a cabo esta importante etapa^(10)^.

Así, considerando la relevancia de enfermeros y médicos en los servicios de salud y la importancia de que el tema sea trabajado en cursos de pregrado, este estudio buscó conocer las contribuciones del uso de la simulación clínica con estudiantes de pregrado de salud, para el cuidado de mujeres adolescentes en situación de violencia de pareja en el contexto de la Atención Primaria de Salud. Se entiende que este abordaje está en consonancia con las perspectivas de fortalecimiento del trabajo de enfermería y salud para atender a las necesidades de las adolescentes, especialmente en el contexto de la APS, a través de acciones culturalmente sensibles y con perspectiva de género. Además, puede proporcionar una herramienta para instrumentalizar a profesores y enfermeros en el cuidado de la salud de esta población^(11)^.

## Método

### Tipo o diseño del estudio

Estudio descriptivo-exploratorio con enfoque cualitativo^(12)^, que siguió el marco conceptual del *debriefing* holístico^(10)^. Los aspectos contenidos en los *Consolidated Criteria for Reporting Qualitative Research* (COREQ) guiaron el diseño y la presentación de esta investigación.

### Local y entorno del estudio

El sitio de estudio fue una universidad pública situada en el interior del estado de São Paulo, en Brasil, que tiene 254.857 habitantes en un área territorial de 1.137 km^2^. La universidad tiene 64 programas de pregrado y 15.518 estudiantes estaban matriculados en 2023. La muestra fue elegida por conveniencia de los investigadores (ya tenían contacto con los profesores y estudiantes) y porque la universidad utiliza estrategias de simulación en sus programas de pregrado.

### Período

La recogida de datos tuvo lugar entre agosto y octubre de 2023.

### Criterios de selección y participantes

Los criterios de inclusión de los participantes fueron: ser estudiante de pregrado de medicina o enfermería en la universidad de estudio; tener más de 18 años; haber tenido contacto con asignaturas teóricas que tratasen sobre violencia o atención a niños, adolescentes y mujeres; presentarse el día y hora acordados para la actividad de simulación y participar en todas las etapas propuestas. El criterio de exclusión fue ausentarse de las actividades universitarias por cualquier motivo.

Para la selección de los participantes, la investigación contó con el apoyo de la Coordinación de Comunicación Social de la Universidad, a través de correos electrónicos enviados a los coordinadores de los cursos, así como de la publicación electrónica en las plataformas y redes sociales de los Centros Académicos de la Universidad. No hubo contacto con los investigadores en esta etapa.

Los participantes fueron aquellos que contactaron con la investigadora y se presentaron los días previstos para la simulación, en la Unidad de Simulación Sanitaria, que cuenta con entornos simulados muy similares a los consultorios de APS. Todos los estudiantes que se presentaron aceptaron participar en la investigación.

### Recogida de datos

La simulación clínica se guio por el escenario “Violencia de pareja contra mujeres adolescentes en Atención Primaria de Salud”, basado en la Taxonomía de Bloom para la construcción de los objetivos de los procesos educativos basados en los dominios cognitivo, procedimental y actitudinal; las normas de práctica recomendadas para el diseño de simulaciones por la *International Nursing Association for Clinical Simulation and Learning* ^(13)^; y el guión para la construcción de un escenario simulado^(14)^. Para finalizar el escenario se utilizó la guía “*Three stages of holistic debriefing*”^(10)^. El escenario fue elaborado y validado por 26 jueces especializados en simulación clínica y violencia.

Las simulaciones con estudiantes de medicina y enfermería tuvieron lugar por separado y el objetivo general de aprendizaje del escenario era que el estudiante tuviera, al final de la actividad, la capacidad de desarrollar cuidados a mujeres adolescentes en situación de VP.

La sesión informativa previa (*pre-briefing*) se llevó a cabo mediante el envío de un folleto informativo sobre la violencia contra las mujeres^(15)^ y una infografía sobre la violencia de género entre los adolescentes^(3)^. La sesión informativa (*briefing*), que duró entre 10 y 15 min, comenzó con una presentación del entorno, el equipo, los recursos, la presentación de los facilitadores, información para los estudiantes sobre el objetivo de aprendizaje que se alcanzaría en el escenario simulado, así como el contrato de trabajo, como el respeto mutuo, la confidencialidad y la ética. A los estudiantes se les entregó un Formulario de Consentimiento Libre e Informado, respondieron un cuestionario sobre su perfil socioeconómico y una descripción del caso clínico, que fue leída al grupo. El día de la simulación clínica, los estudiantes fueron informados sobre la formación de los investigadores y el motivo de la realización del trabajo. Además, si no aceptaban participar en la investigación, podían igualmente participar en la simulación.

A continuación, se eligió una pareja de estudiantes para asistir a la adolescente en situación de violencia, para participar en el escenario, y a su madre, con una duración máxima de 30 min. Los demás estudiantes permanecieron como espectadores del escenario en un espacio separado en la propia unidad de simulación.

Los escenarios fueron dirigidos por dos mujeres cisgénero facilitadoras, ambas enfermeras e investigadoras en una universidad pública, una estudiante de postdoctorado y la otra profesora. Ambas expertas en el tema disponían de un flujograma que les ayudaba a conducir las actividades, buscando alcanzar los objetivos de aprendizaje inicialmente propuestos. En el escenario de la investigación no participaron más personas que las descritas: investigadores facilitadores, estudiantes y actores.

El *debriefing* se grabó con una aplicación de grabación de voz para teléfonos móviles y se transcribió manualmente. El debate se llevó a cabo utilizando un instrumento ideado por los investigadores, basado en las “Tres etapas del *debriefing* holístico”^(10)^, a saber: (1) Focalizado (autorreflexión inmediata de los estudiantes tras la experiencia práctica), con las siguientes preguntas: “¿Cómo te sientes sobre la experiencia de simulación? ¿Qué has aprendido al participar en la experiencia de simulación? ¿Qué habilidades crees que has desarrollado durante la experiencia de simulación?”; (2) Formativa (*debriefing* continuo con el grupo más amplio a lo largo de la simulación) con las siguientes preguntas: “Como grupo, ¿cómo te has sentido durante la simulación? Como grupo, ¿qué creen que saben/entienden mejor ahora? Como grupo, ¿creen que esta experiencia de simulación y el debate han ayudado a desarrollar las habilidades psicomotoras? ¿Por qué sí o por qué no? ¿Qué has aprendido durante esta simulación que sea similar a lo que aprendiste en la carrera? ¿Qué es diferente?”; y (3) Sumativa (reflexión final con el grupo más amplio y su aplicación al aprendizaje obtenido), con las preguntas: “¿Cómo te sentiste después de la experiencia de simulación? ¿Cómo percibiste la progresión de tu aprendizaje a través de esta experiencia? ¿Qué tipo de habilidades crees que has desarrollado a través de la experiencia de simulación? ¿Cree que la experiencia y los conceptos de violencia contra los adolescentes que discutimos en la sesión informativa previa son claros para usted, basándose en la evidencia de las mejores prácticas? Por favor, justifique su respuesta. ¿Cree que la actividad de simulación contribuyó a su actuación profesional en el trato con adolescentes en situaciones de violencia de pareja?”.

Se realizó una prueba piloto inicial para comprobar la idoneidad del instrumento de recogida de datos, y no fue necesario introducir cambios; los datos de la prueba piloto se incorporaron a la investigación. No se repitieron los grupos y la saturación de los datos, es decir, la profundización en los elementos que respondían a las preguntas de la investigación se alcanzó en el quinto grupo focal, con dos más ya programados. Inmediatamente después de la simulación clínica, el investigador principal tomó notas sobre las simulaciones en un diario de campo, lo que ayudó a la discusión de los resultados.

En total, se realizaron siete simulaciones con grupos de aproximadamente ocho estudiantes, en las que participaron 28 estudiantes de pregrado de enfermería y 30 de medicina. Teniendo en cuenta todas las etapas, la actividad más corta duró 2h35min y la más larga 2h55min.

Para la presentación de los datos, se identificó a los participantes según el curso al que pertenecían, *Med* para Medicina y *Enf* para Enfermería, seguido de un número secuencial, según el orden en que hablaron durante la actividad.

### Tratamiento y análisis de los datos

Los datos sobre el perfil de los participantes, recogidos mediante el cuestionario, se presentaron utilizando estadísticas descriptivas. Los datos del *debriefing* se transcribieron íntegramente mediante la aplicación *Google Documents* y se analizaron mediante la técnica de análisis temático reflexivo e inductivo, es decir, emergente de los datos^(16)^. Se generaron 16 códigos iniciales y cinco intermedios (“La complejidad de la violencia”, “Sentimientos y emociones implicados en la atención”, “La importancia de la acogida y el vínculo”, “Las especificidades de la atención a casos de violencia contra adolescentes” y “Habilidades desarrolladas a través del uso de la simulación”), que a su vez se agruparon en dos temas finales.

Para hacer este proceso más confiable, se siguieron los siguientes criterios: (1) el uso de un diario de campo, que permitió el registro cuidadoso y completo del proceso de recolección y de las inferencias en el campo; (2) la construcción de códigos y temas entre dos investigadores (1º y 2º autor), acordados por un tercero (último autor); (3) la revisión de los datos interpretados con los participantes para verificar el significado o la necesidad de nuevas inserciones - se hicieron 17 *feedbacks* por correo electrónico, y no se incorporó ninguna información nueva.

### Aspectos éticos

La investigación fue aprobada por el Comité de Ética en Investigación de la Universidad Federal de São Carlos, bajo CAAE 63438122.1.0000.5504.

## Resultados

De los 58 estudiantes, la mayoría tenía entre 20 y 25 años (medicina n=25, 83%; enfermería n=21, 75%). En cuanto al color, la mayoría se declaraba blanca (medicina n=22, 73%; enfermería n=19, 68%). Todos eran cisgénero, con 17 estudiantes de medicina declarándose hombres cis (57%) y 13 (43%) mujeres cis. Entre los estudiantes de enfermería, tres (11%) se declararon hombres cis y 25 (89%) mujeres cis.

Después de analizar los datos cualitativos, los resultados se dividieron en dos temas finales: “La complejidad de la violencia” y “Competencias desarrolladas mediante el uso de la simulación”.

### La complejidad de la violencia

Este tema abordó la complejidad de los elementos que intervienen en el tratamiento de los casos de VP contra mujeres adolescentes. El primer aspecto se refería a la presentación de la situación, que abarca aspectos familiares más amplios que es necesario comprender para proporcionar una atención integral. La presencia de la madre en la consulta inicial, así como los conflictos familiares, incluidas las sospechas de violencia doméstica, dificultaron el abordaje:

*Y también explorar la cuestión de la bebida del padre, que es tal vez una cuestión de un alcohólico en el entorno familiar, por lo que sería posible buscar esta información un poco más también*. (Med 28)

*Entonces también me interesaría hablar sólo con la madre, está todo el tema familiar, el entorno familiar, porque ella huye de ese entorno. No estaría bien hablar de eso delante de ella.* (Enf 5)

*Hay dos personas activas en el atendimiento, con historias distintas. Así que a veces, cuando asistimos, es sólo una persona o la otra, cuando hay dos, está allí complementando la historia de la otra persona. Esta es la primera vez que tenemos dos personas con puntos de vista diferentes sobre el mismo tema.* (Med 21)

*Me pareció muy complejo, porque está la cuestión del padre, no sabía si podía preguntar «¿tu padre te pegaba? ¿Ha habido otro tipo de violencia?». O si es testigo de otras violencias con su madre [...] está la cuestión del novio, son muchas cuestiones complejas en un solo ser.* (Enf 1)

Como se muestra en la última intervención, se revelaron las particularidades de la vivencia de la VP en la adolescencia. A los participantes les resultó más difícil abordar y tratar el caso, dado que el adolescente no acudía a la unidad por una necesidad explícita.

*Es una adolescente y es un poco tímida.* (Med 5).

*Ella no quería estar aquí, no quería estar en la cita, fue obligada por su madre, así que ya estaba desinteresada por todo. Estaba aquí porque su madre la obligó.* (Enf 8)

La ambivalencia en la relación, estar enamorada y una vivencia más velada de violencia - dada por los celos y el control, surgieron en los informes como aspectos desafiantes.

*Ella tampoco se veía en una situación de violencia, ¿no? Así que eso fue mucho más desafiante.* (Enf 17)

*Porque tenemos que recordar la cuestión de la madurez, del desarrollo. Así que, a su edad, todavía no tiene suficiente madurez de desarrollo para comprender plenamente y hacer un juicio sobre lo que está bien y lo que está mal.* (Med 4)

*Al principio dijo que echaba de menos salir con sus amigas, que su novio se lo prohibía y la chantajeaba, y luego cuando le preguntamos por su red de apoyo dijo que sólo tenía a su novio y que el resto era todo falso.* (Enf 15)

*Y por lo que parece, es una persona muy tímida, hasta en la forma de sentarse y demás. Así que es muy difícil que se te ocurra una idea, decir algo, cuando ella depende mucho de su novio, cierto, entre otras cosas porque no puede lidiar con sus propios padres.* (Med 6)

De la última afirmación se desprende que los participantes tuvieron dificultades para entablar un diálogo con un perfil adolescente, que suele ser menos elaborado verbalmente en el primer contacto. La falta de contacto con los adolescentes durante su carrera académica en los servicios de salud supuso mayores retos a la hora de establecer una relación:

*Es mucho más difícil, creo, para nosotros establecer un vínculo con un adolescente, que a veces sólo tiene más contacto con sus padres, con sus amigos, ¿no?* (Enf 19)

*Me pareció un poco más complicado de tratar, porque ahora estamos tratando con un adolescente, algo con lo que nunca habíamos tratado antes*. (Med 12)

El tema de “ser menor de edad” fue recurrente en la discusión de la situación vivida, especialmente debido a las dudas sobre la denuncia legal, el mantenimiento del secreto y de los vínculos, las relaciones de poder establecidas entre el adolescente y el adulto y el mejor manejo del caso:

*Yo tenía muchas dudas sobre eso, sobre cómo decir que no era normal que una niña de 13 años tuviera una relación con un adulto de veinte. No sabría cómo decirlo. ¿No es un delito?* (Med 2)

*En este caso, ¿sería sólo el ayuntamiento o habría que contactar con otro organismo? ¿Quizás la escuela? ¿Cómo se comunica la Comisaría de la Mujer con esta red?* (Med 6)

*Este tema de los celos, cierto, la diferencia en la relación de poder entre un adulto de 21 años y una adolescente de 13. E incluso hay algunas otras cosas que mencioné, como si tiene miedo de ir en contra de su novio, porque quería explorar más este equilibrio de poder.* (Enf 17)

*Hablar más con la paciente, hacerle entender que esto es un problema, no hablar de la queja enseguida, si no, no volverá jamás.* (Enf 7)

### Competencias desarrolladas mediante el uso de la simulación

En este tema, las competencias que los estudiantes adquirieron o ejercitaron a través de la simulación surgieron en términos de conocimientos, habilidades y actitudes. Debido al objetivo particular de la simulación, es importante destacar que el tratamiento de la violencia, especialmente contra los adolescentes, se considera un “*tema tenso y pesado* (Enf 12)”, que hace aflorar sentimientos de los que es necesario hablar y tratar:

*Para ser sincera, sentí mucha incomodidad. Porque es una situación muy común, que me recordaba a amigas mías, que yo veía cómo hablaban y todo, por lo que pasan, cómo se sienten, entonces me trae esa incomodidad y esos recuerdos.* (Enf 23)

*Así que me angustia vivir un caso de violencia, sobre todo siendo ella una adolescente menor de edad.* (Med 3)

*Creo que es muy difícil, incluso me negué a hacer el simulacro porque no me sentía cómoda. Y no es cuestión de entender y aceptar, no me siento cómoda haciéndolo. Así que prefiero observar, ver cómo lo hace la gente para intentar aprender y sacar algo de ello*. (Med 7)

La última afirmación demuestra la coherencia de la utilización de la estrategia de simulación para facilitar el proceso de enseñanza-aprendizaje, tornando el tema más viable y palpable. En ese sentido, los participantes relataron haber movilizado conocimientos ya adquiridos, como la importancia de la acogida, del vínculo y de la continuidad de los cuidados, atributos esenciales de la APS, además de articular aspectos teóricos sobre la violencia y la red de apoyo:

*Creo que en la primera consulta tuvimos que acoger, mostrar que es un ambiente seguro y que estábamos allí para escuchar sin juzgar*. (Enf 2)

*Ella creó un vínculo con los pacientes, para no acusar a nadie ni hacer que nadie se sintiera mal, con el fin de tener esta continuidad de cuidados. Creo que eso es lo peor de la APS: espantas al paciente en la primera visita y el no vuelve más.* (Med 14)

*Una cosa a la que acabé volviendo es que, cuando estudiábamos la violencia familiar y doméstica, las personas que han vivido en una casa donde hay violencia tienen más tendencia a perpetuar ese escenario violento. Y eso es lo que pasó en este caso: la madre venía de una familia con violencia, vive en un escenario de violencia, y aparentemente su hija también está entrando en un escenario de violencia. Entonces, creo que explorar cómo la hija ve el caso de su madre puede ayudarla a reflexionar sobre cómo ella también está entrando en una situación, tal vez similar, y puede llevar al mismo futuro.* (Enf 6)

*Averigua si tiene amigos, algún otro familiar cercano, para intentar ver su red de apoyo. Con más tiempo, quizá como forma de vinculación, remítala a un psicólogo de la unidad, a un grupo de terapia. ¿Por qué no tiene amigos en la escuela? ¿Qué pasó, si pasó algo, si su novio la aleja de esas amistades?* (Med 15)

En este sentido, los estudiantes fueron capaces de practicar la atención a la VP entre adolescentes en APS; aspectos revelados para esta habilidad fueron la visión holística, más allá de los signos y síntomas presentes, la escucha activa y la mediación de conflictos. Se mencionaron aspectos actitudinales sin “receta” como el respeto y el tono de voz:

*Creo que también hay otro punto que me olvidé de mencionar, que es la escucha activa. Es decir, que el adolescente hable y tú prestes atención a lo que dice, la mires [...]. Ya había leído en algunos libros que el hecho de que te quedes callado un poco más hace que la persona diga algo nuevo que quizá no diría si la interrumpieras con una pregunta en ese momento*. (Med 9)

*Bueno, creo que es todo un reto, porque a veces nos preocupamos por otras cosas que estamos intentando identificar y acabamos pasando por alto estas señales y si está pasando algo más*. (Enf 1)

*De lo que me he dado cuenta es de que necesito aprender cómo abordar este caso, la forma de hablar, el tono de voz, todavía me cuesta un poco manejar estos casos.* (Enf 2)

Con la posibilidad de simular un atendimiento, la atención a la VP contra mujeres adolescentes en APS fue entendida como un proceso que requiere tecnologías livianas, tiempo y acciones prolongadas. Este elemento facilitó la problematización de las discusiones sobre el proceso de denuncia, así como el equilibrio necesario en estas situaciones:

*Es una situación complicada, cierto, no puedes decirle simplemente: “mira, rompe con tu novio, porque...”, no puedes decir eso. Yo creo que lo más seguro sería que ella entendiera las repercusiones de esa relación en su vida, y para eso sería un proceso, no sería en una consulta, sería mucho tiempo de hablar, tanto con los profesionales de la salud como con la madre y el padre*. (Med 4)

*Esta es una habilidad que tengo que desarrollar, porque quiero saberlo todo sobre el paciente de inmediato. Ya pienso “salga de esto, mujer, que te están oprimiendo”.* (Enf 12)

Por último, los participantes destacaron la relevancia de la simulación, situándolos como personas activas en la construcción del proceso de enseñanza-aprendizaje. El *debriefing* se señaló como una etapa importante para dar sentido a lo aprendido, así como para adquirir nuevas competencias a través del aprendizaje significativo:

*Me parece genial la simulación, porque vemos la teoría, los signos, y ustedes nos lo pones fácil, incluyendo casi todos. Así, la persona evita hablar, no te mira a la cara para contestar, el conflicto que puede haber entre la madre y la niña, en fin. Esto es bueno porque nos enfatiza que va a pasar, nosotros no sólo leemos lo que está en la teoría.* (Enf 16)

*Entonces, yo creo que la simulación ha contribuido mucho a nuestra comprensión de los adolescentes, sus ansiedades, demandas y todo lo demás.* (Med 27)

*También me pareció estupendo que pudiéramos hacer preguntas, porque como habíamos mencionado, este tema de la notificación o denuncia obligatoria, llevar un registro de ello, no lo habíamos visto en profundidad, me pareció interesante.* (Med 7)

## Discusión

Los estudiantes percibieron la complejidad de tratar casos de VP contra mujeres adolescentes en el contexto de la APS debido a los amplios aspectos familiares; la presencia de la madre durante el proceso; las particularidades de la VP en la adolescencia; la dificultad del diálogo en este período; la percepción de la ambivalencia de los sentimientos en una relación violenta y el equilibrio entre la preservación de la autonomía de la persona adolescente y la obligación de denunciar el caso. A pesar de esta complejidad, de tener que enfrentarse a los sentimientos angustiosos que despierta el hecho, los estudiantes desarrollaron diversas competencias, como la capacidad de mediar en los conflictos y actitudes como el respeto, identificando que la atención a la VP se establece como un proceso. Para desarrollarlo, se movilizaron atributos de la APS, como la necesidad de continuidad del cuidado a través del vínculo, la acogida y el reconocimiento de la red de apoyo.

El perfil sociodemográfico de los participantes corrobora un estudio en el área^(17)^, en el cual la mayoría de los estudiantes universitarios del área de salud tiene hasta 25 años, es blanca y formada por mujeres cis. La atención a adolescentes en situación de violencia tiene muchas especificidades^(3)^. Durante la simulación, se repitieron las dudas sobre la diferencia entre notificación y denuncia y los límites de la autonomía del adolescente. Todos los casos de violencia son de notificación obligatoria - se trata de una comunicación obligatoria a la autoridad sanitaria, realizada por profesionales, sobre la ocurrencia de problemas específicos sospechados o confirmados, que permite analizar los datos y puede servir de apoyo para la toma de decisiones^(18)^. Este aspecto es diferente de la comunicación e incluso de la articulación de la atención a los adolescentes, que a veces se descuida. Considerar la atención a los adolescentes más allá de los prismas convencionales de salud (basados en factores de riesgo y protección), e incluirlos como personas socialmente activas, especialmente los grupos vulnerables, se considera un motor para una participación significativa en la acción global acelerada para la salud de esta población^(19)^.

En Brasil, según la Ley 8069 del Estatuto del Niño y del Adolescente (ECA, en la sigla en portugués), los adolescentes son sujetos de derechos que pueden ser atendidos de forma autónoma en los servicios de salud^(20)^. Teniendo esto en cuenta, los estudiantes consultaron a la adolescente sobre si quería o no que su madre se fuera para escucharla a solas durante la simulación. Además, aunque no haya nada específico en el ECA que prohíba el noviazgo entre un adolescente y un adulto, el artículo 217-A del Código Penal brasileño tipifica como delito la conjunción carnal o el acto libidinoso con una persona menor de 14 años, lo que conlleva una pena de prisión de entre ocho y 15 años^(21)^. Según el ECA, es obligatorio denunciar ante el Consejo Tutelar las sospechas o confirmaciones de violencia contra niños y adolescentes. Aunque la denuncia sea obligatoria, pues puede estar ocurriendo un crimen, durante la atención en APS es necesario reflexionar sobre este proceso, incluso con la adolescente, así como establecer un cuidado extendido para ella. Es necesario comprender la dinámica familiar, con la adolescente en el centro de esta atención, en consonancia con el ECA y otras legislaciones^(20-22)^.

Aún en relación con la atención prestada en la simulación, los participantes manifestaron dudas sobre cómo abordar a la adolescente y si podían interrogarla directamente sobre la violencia. Investigadores de una universidad norteamericana han elaborado una guía para ayudar a los estudiantes de enfermería a reconocer y ayudar a los adolescentes que sufren violencia en el noviazgo. Destacaron la importancia de preguntar directamente sobre este hechos, ya que muchos adolescentes tienen miedo de contar estos problemas o pueden no percibir el comportamiento de su pareja como violento^(23)^.

Un estudio analizó los currículos de pregrado de enfermería en instituciones públicas de Brasil en relación con el abordaje de la violencia contra la mujer^(24)^. Se identificó que el abordaje de la salud del adolescente se refiere más a aspectos de violación de los derechos sexuales y reproductivos, con preocupación relacionada al embarazo en la adolescencia^(24)^. Este hallazgo refuerza la importancia de la simulación de la violencia contra una mujer adolescente para los estudiantes de pregrado, ya que es un abordaje que va más allá de los aspectos biológicos relacionados con la violencia y está poco desarrollado en los currículos de pregrado. Esto también está en consonancia con la importancia de este tema para prevenir la violencia de género en el futuro.

Teniendo en cuenta la importancia de preparar a los estudiantes, especialmente de medicina, enfermería y obstetricia, para atender a mujeres en situaciones de violencia, la Organización Mundial de la Salud (OMS)^(22)^ ha elaborado un documento que ofrece directrices para la formación profesional, considerando que abordar la violencia contra las mujeres en los cursos de pregrado puede ayudar a los estudiantes a sentirse más cómodos en el futuro a la hora de tratar estos casos. El documento enfatiza que los estudiantes deben desarrollar habilidades apropiadas, tales como cuándo y cómo hacer preguntas; la mejor manera de responder a las mujeres; qué respuestas emocionales pueden afectar la atención; cómo adoptar una postura que no culpe ni juzgue; cómo recoger rastros (cuando sea necesario); conocer las leyes y los servicios básicos para la atención, entre otros^(22)^. La simulación demostró estar en consonancia con las directrices de la OMS, ya que los estudiantes informaron de diversas competencias desarrolladas a través de la práctica, tanto procedimentales como actitudinales, como el enfoque holístico, la mediación de conflictos, la escucha activa y el respeto.

En el contexto del desarrollo de competencias actitudinales, los investigadores analizaron los protocolos disponibles para orientar a los profesionales sobre la VP en el sistema sanitario español, realizando grupos con médicos en ejercicio para ver cómo se utilizaban estos instrumentos en la práctica. Los profesionales refirieron sentimientos de miedo, frustración o impotencia como obstáculos para abordar el problema de la VP, y es fundamental que exista formación para que las respuestas emocionales de los profesionales no influyan negativamente en la atención a las mujeres^(25)^. En nuestra investigación, encontramos que estos sentimientos aparecen y pueden ser manejados desde el pregrado, por lo que es fundamental que este tema sea trabajado con los estudiantes.

Los estudiantes de medicina y enfermería que participaron de la simulación demostraron la importancia de acoger y establecer un vínculo con el adolescente, identificando estos como atributos fundamentales del trabajo en APS. El proceso de trabajo en las unidades de APS en Brasil debe priorizar el establecimiento de vínculos y la acogida entre los equipos y la población, que deben estar presentes en todas las relaciones de atención^(6),^ 26.

En esta investigación, los datos cualitativos se recogieron durante el *debriefing*. Nuestros resultados demostraron la importancia y la riqueza de la información, la reflexión y el aprendizaje que esta fase de la simulación proporcionó a los estudiantes. Este aspecto se ve corroborado por una revisión bibliográfica que analizó 140 estudios en los que se destacaba la importancia del *debriefing* para la enseñanza y el aprendizaje en la formación de enfermería^(27)^. Esta revisión destaca la relevancia del *debriefing* holístico, que permitió articular la adquisición de conocimientos, habilidades y actitudes en sus ámbitos formativo y sumativo al abordar eventos complejos.

Investigadores de una universidad alemana implementaron un curso electivo para estudiantes de medicina sobre violencia doméstica y destacaron, en sus conclusiones, la importancia de que el tema fuera abordado en los cursos de pregrado, ya que los participantes presentaban lagunas en sus conocimientos^(17)^. Esos datos corroboran los encontrados en este estudio, destacando una vez más la importancia de esa actividad para la formación de futuros profesionales de forma significativa.

Las limitaciones de este estudio incluyen el hecho de que se trataba de una muestra específica de estudiantes de una universidad pública en sus últimos años de estudios de licenciatura en medicina y enfermería. No se incluyeron estudiantes del sector privado, de otras carreras de salud ni de otros programas de pregrado. Además, no fue posible realizar las simulaciones de forma interprofesional debido a las diferencias de horarios disponibles entre los estudiantes.

A pesar de sus limitaciones, este estudio demostró cómo la simulación de la atención a la VP de una adolescente en APS es una poderosa herramienta para el proceso de enseñanza-aprendizaje en cursos de pregrado en salud. La mirada cualitativa del proceso también permitió comprender que esa estrategia puede ser coherente con temas complejos que involucran la adquisición y experimentación de competencias cognitivas, procedimentales y actitudinales. Sugerimos que las simulaciones sean realizadas con estudiantes de universidades públicas y privadas y de otros cursos de pregrado en salud, de forma interprofesional.

## Conclusión

Retomando el objetivo de este estudio, el uso de la simulación clínica ha hecho contribuciones relevantes al proceso de enseñanza-aprendizaje de estudiantes de pregrado en salud sobre la atención a mujeres adolescentes en situación de VP en el contexto de la APS. Hubo reflexiones sobre las especificidades de la atención a mujeres adolescentes en situaciones de VP, como la diferencia entre notificación y denuncia y la autonomía de las adolescentes durante los atendimientos. También se favoreció el desarrollo de nuevas competencias procedimentales y actitudinales, como el abordaje holístico, la mediación de conflictos, la escucha activa, el respeto y la importancia de preguntar directamente sobre la ocurrencia de violencia. Así, los atributos de la APS fueron movilizados e incorporados, permitiendo a los estudiantes estar mejor preparados para la práctica profesional cuando se encuentren con esas situaciones, contribuyendo para una mejor atención.
